# Fatty acid synthase: a novel target for antiglioma therapy

**DOI:** 10.1038/sj.bjc.6603350

**Published:** 2006-09-12

**Authors:** W Zhao, S Kridel, A Thorburn, M Kooshki, J Little, S Hebbar, M Robbins

**Affiliations:** 1 Departments of Radiation Oncology and Neurosurgery, Brain Tumor Center of Excellence, Wake Forest University School of Medicine, Medical Center Boulevard, Winston-Salem, NC 27157, USA; 2 Department of Cancer Biology, Wake Forest University School of Medicine, Winston-Salem, NC 27157, USA

**Keywords:** fatty acid synthase, glioma cells, cerulenin, cell cycle arrest, apoptosis, Bcl-2

## Abstract

High levels of fatty acid synthase (FAS) expression have been observed in several cancers, including breast, prostate, colon and lung carcinoma, compared with their respective normal tissue. We present data that show high levels of FAS protein in human and rat glioma cell lines and human glioma tissue samples, as compared to normal rat astrocytes and normal human brain. Incubating glioma cells with the FAS inhibitor cerulenin decreased endogenous fatty acid synthesis by approximately 50%. Cell cycle analysis demonstrated a time- and dose-dependent increase in S-phase cell arrest following cerulenin treatment for 24 h. Further, treatment with cerulenin resulted in time- and dose-dependent decreases in glioma cell viability, as well as reduced clonogenic survival. Increased apoptotic cell death and PARP cleavage were observed in U251 and SNB-19 cells treated with cerulenin, which was independent of the death receptor pathway. Overexpressing Bcl-2 inhibited cerulenin-mediated cell death. In contrast, primary rat astrocytes appeared unaffected. Finally, RNAi-mediated knockdown of FAS leading to reduced FAS enzymatic activity was associated with decreased glioma cell viability. These findings suggest that FAS might be a novel target for antiglioma therapy.

In 2006, 18 820 individuals living in the US will be diagnosed with primary brain cancer (Jemal *et al*, 2006). The most common and most lethal primary brain tumour in adults is glioblastoma multiforme. Glioblastoma multiforme represents one of the most challenging of all cancers to treat successfully, characterised not only by aggressive proliferation and expansion but also by inexorable tumour invasion into distant brain tissue (Kew and Levin, 2003). Improvements in multimodality treatments including surgery, radiotherapy, and the recent addition of temozolomide have improved median survival, but this is still only approximately 14 months (Stupp *et al*, 2005) and long-term survival is dismal. The development of novel biological therapies for primary brain tumours remains an urgent need. Current cancer treatment is rapidly evolving in parallel with an understanding of tumour biology (Weiss, 2000; Karpati and Nalbantoglu, 2003). Genetic abnormalities and differential gene expression between normal and cancer cells can provide novel targets for anticancer therapy (Lang *et al*, 1999; Bansal and Engelhard, 2000; Grill *et al*, 2001).

Fatty acid synthase (FAS) is a 270 kDa cytosolic multifunctional polypeptide and is the primary enzyme required for catalysing the conversion of dietary carbohydrates to fatty acids (Wakil, 1989). Normal cells preferentially use circulating dietary fatty acids for the synthesis of new structural lipids. Thus, FAS expression is generally low to undetectable in normal human tissues, other than the liver and adipose tissue. In contrast, FAS is overexpressed in many human tumours, including carcinoma of the breast (Milgraum *et al*, 1997; Wang *et al*, 2001b), prostate (Epstein *et al*, 1995; Swinnen *et al*, 2002), colon (Rashid *et al*, 1997), ovary (Gansler *et al*, 1997), endometrium (Pizer *et al*, 1998b), mesothelium (Gabrielson *et al*, 2001), lung (Piyathilake *et al*, 2000), thyroid (Vlad *et al*, 1999), and stomach (Kusakabe *et al*, 2002). Abnormally active endogenous fatty acid metabolism appears to be important for cancer cell proliferation and survival (Ookhtens *et al*, 1984). Moreover, overexpression of FAS in breast, prostate, and thyroid cancers has been associated with more aggressive malignancies (Epstein *et al*, 1995; Gansler *et al*, 1997; Vlad *et al*, 1999; Swinnen *et al*, 2002). The preferential expression of FAS in cancer cells suggests that FAS could be a promising target for antitumour therapy (Kuhajda, 2006).

Inhibitors of FAS have been used to study the loss of FAS function in tumour cells. The first identified ‘specific’ inactivator of FAS, cerulenin, (2*R*, 3*S*)-2,3-epoxy-4-oxo-7,10-trans,trans- dodecadienamide, is a natural antibiotic product of the fungus *Cephalosporium ceruleans* (Omura, 1976). Cerulenin irreversibly inhibits FAS by binding covalently to the active site cysteine thiol in the *β*-ketoacyl-synthase domain (Funabashi *et al*, 1989). Cerulenin is selectively cytotoxic to a number of established human cancer cell lines, including breast (Gabrielson *et al*, 2001), colon (Pizer *et al*, 1998a), and prostate (Furuya *et al* 1997; Pizer *et al*, 2001). Kuhajda *et al* (2000) reported that cerulenin inhibited fatty acid synthesis in tumour cells in a dose-dependent manner; the cytotoxic effect of cerulenin to human breast tumour cells generally paralleled the level of endogenous fatty acid synthesis. Fatty acid synthase inhibition by cerulenin leads to apoptotic cell death in breast, prostate, and colon cancer cells (Furuya *et al*, 1997; Huang *et al*, 2000; Li *et al*, 2001). However, cerulenin's chemical instability renders it inappropriate as a systemic anticancer agent. C75, a potent derivative of cerulenin and a more stable form of FAS inhibitor, has been tested recently for its anti-breast tumour effects (Kuhajda *et al*, 2000; Pizer *et al*, 2000). *In vivo* and *in vitro* studies have confirmed the selective toxicity of C75 against tumour cells. C75-mediated inhibition of FAS increases malonyl-CoA levels and inhibits CPT-1 activity, preventing the oxidation of newly synthesised fatty acids. High levels of malonyl-CoA and CPT-1 inhibition might represent a mechanism, whereby FAS inhibition leads to tumour cell death (Pizer *et al*, 2000). C75 treatment of mesothelioma and prostate cancer xenografts in nude mice led to significant inhibition of tumour growth (Gabrielson *et al*, 2001; Pizer *et al*, 2001). Subcutaneous xenografts of MCF7 breast cancer cells in nude mice treated with C75 showed fatty acid synthesis inhibition, apoptosis, and inhibition of tumour growth to less than 12.5% of control volumes, without comparable toxicity in normal tissues (Pizer *et al*, 2000).

More recently, an activity-based proteomics strategy revealed *in vitro* and *in vivo* antitumour activity for Orlistat, an FDA-approved drug used for treating obesity. Orlistat is a novel inhibitor of the thioesterase domain of FAS that inhibited prostate cancer cell proliferation, induced apoptosis, and inhibited the growth of PC-3 human tumour xenografts implanted in nude mice (Kridel *et al*, 2004).

To date, there is little information regarding FAS expression in brain tumours. Slade *et al* (2003) reported that significantly larger amounts of FAS protein were detected in the neuroblastoma cell line SK-N-SH compared with that in the human fibroblast cell line Hs27. We hypothesised that FAS would be highly expressed in glioma cells and that inhibition of FAS would lead to glioma cell death. In this paper, we show that this hypothesis appears to be correct. For the first time, we show upregulation of FAS in glioma cells and human glioma tissue, dose-and time-dependent decreases in glioma cell viability, and clonogenic survival with FAS inhibition, as well as increased apoptotic cell death. These studies identify FAS as a potential target for glioma therapy.

## MATERIALS AND METHODS

### Cell culture

Human U373, U118, U87, and U138 as well as rat C6 glioma cell lines were obtained from the American Type Culture Collection (Rockville, MD, USA). The ethyl nitrosourea-induced rat 36B10 glioma cell line was obtained from Dr Vincent Traynelis, Division of Neurosurgery, Department of Surgery, University of Iowa (Spence & Coates, 1978) and routinely maintained in high glucose DMEM containing 5% bovine calf serum, 2 mM L-glutamine, 100 IU ml^−1^ penicillin, 100 *μ*g ml^−1^ streptomycin, and 0.1 mM non-essential amino acids (all from GIBCO, Gaithersburg, MD, USA) at 37°C with 5% CO_2_ in air. Cells were trypsinised and reseeded at a 1:5 dilution every 3 days. Human U251 and SNB-19 glioma cell lines were a kind gift of Dr Sue Hess (Department of Radiation Oncology, WFUSM) and were maintained in DMEM/F12 and F10 media supplemented with 10% foetal bovine serum (FBS), 2 mM L-glutamine, 100 IU ml^−1^ penicillin, and 100 *μ*g ml^−1^ streptomycin. Primary rat astrocytes were isolated from 1- to 2-day-old Sprague–Dawley rat pups as described previously (Murphy, 1990). Cortices were removed from the rat pups; the meninges were stripped and homogenised. After incubation with trypsin for 15 min in a 37°C shaking water bath, the homogenate was centrifuged and the pellet was resuspended in a trypsin inhibitor/DNAse solution, triturated, and layered over a bovine serum albumin solution. The cell pellet was resuspended and maintained in MEM medium with 1% L-glutamine, 10% FBS, and 6g l^−1^ glucose at 37°C in a humidified atmosphere of 5% CO_2_ until ready to use.

### Human glioma and normal brain tissue

Human brain tissue lysates were prepared by homogenisation in modified RIPA buffer (150 mM sodium chloride, 50 mM Tris-HCl (pH 7.4), 1 mM phenyl methyl sulphonyl fluoride, 1% Triton X-100, 1% sodium deoxycholic acid, 0.1% sodium dodecyl sulphate (SDS), 5 *μ*g ml^−1^ aprotinin, and 5 *μ*g ml^−1^ leupeptin). The tissue lysates were centrifuged for 10 min at 10 000 r.p.m. to remove debris and the supernatants were stored at −80°C until use.

### Fatty acid synthesis

To test whether cerulenin inhibits FAS activity in glioma cells, lipid synthesis was determined using ^14^C-labelled acetic acid methods as described previously (Rashid *et al*, 1997). Briefly, 5 × 10^5^ SNB-19, U251, and C6 cells were plated into 24-well plates for 24 h (three replicates per group). After incubation with 5 *μ*g ml^−1^ cerulenin for 2 h at 37°C, the cells were washed and were then incubated with ^14^C-labelled acetate for 4 h. Total lipids were extracted and ^14^C counts determined using liquid scintillation counting.

### Western blot analysis

Western blot analysis was performed as described previously (Zhao *et al*, 2001). Cells were lysed using lysis buffer containing 50 mM Tris (pH 7.0), 1 mM EDTA, 150 mM NaCl, 1 mM PMSF, 1 *μ*g ml^−1^ aprotinin, 1 *μ*g ml^−1^ leupeptin, 1 mM Na_3_*V*O_4_, and 1 mM NaF, and stored in aliquots at −70°C until use. Ten micrograms of cell lysate was mixed with an equal volume of sample buffer containing 62.5 mM Tris/HCl (pH 6.8), 10% glycerol, 2% SDS, 5% *β*-mercaptoethanol, and 2–3 drops of saturated bromophenol solution, denatured by boiling, and separated in a 7.5% polyacrylamide mini-gel at a constant voltage of 120 V for 2 h. The proteins were transferred by electrophoresis at 100 V for 1 h to ECL nitrocellulose membrane (Amersham, Arlington Heights, IL, USA). The membranes were blocked for 1 h at room temperature in 5% (wt vol^−1^) non-fat dry milk in TTBS containing 20 mM Tris-HCl (pH 7.0), 137 mM NaCl, and 0.05% (vol vol^−1^) Tween-20. After washing in TTBS for 2 × 10 min, the membranes were then blocked with 5% non-fat milk and probed with either primary mouse anti-FAS (Pharmingen, San Diego, CA, USA), mouse anti-Bcl-2 (Santa Cruz, Santa Cruz, CA, USA), or rabbit anti-poly(ADP-ribose) polymerase (PARP) antibody (Athens Biotech, Athens, GA, USA). After washing three times in TBST for 10 min each, the blots were incubated with anti-mouse or anti-goat IgG horseradish peroxidase conjugate (1:10 000 dilution, Sigma, St Louis, MO, USA) for 1 h at room temperature. Antigen was detected using standard chemical luminescence methodology (ECL; Amersham Pharmacia Biotech, Piscataway, NJ, USA).

### Cell viability assay

Cell viability was determined using a modified MTT assay (Griscelli *et al*, 2000). Briefly, 1000–5000 cells well^−1^ were plated in 24-well plates and incubated overnight. Cells were then treated with 0.5–10 *μ*g ml^−1^ cerulenin for 24–144 h. At the end of the follow-up period, MTT in PBS was added and the cultures were incubated for 4 h at 37°C. The dark crystals formed were dissolved by adding to the wells an equal volume of SDS/dimethylformamide (DMF) extraction buffer (20% SDS and 50% N, N-DMF (pH 4.7), in PBS). Subsequently, plates were incubated overnight at 37°C. A 100 *μ*l aliquot of the soluble fraction was transferred into 96-well microplates, and the absorbance at 570 nm was measured using an ELISA plate reader.

### Clonogenic survival assay

Clonogenic survival assays were performed as described previously (Vartak *et al*, 1998). Two hundred and fifty to 2000 cells were plated in 60 mm dishes and incubated overnight. Cells were then treated with 0–10 *μ*g ml^−1^ cerulenin and cultured until colonies had formed (10–14 days). Cells were then fixed for 10 min in a solution containing 10% acetate and 10% methanol. The cells were then stained with 0.4% crystal violet for another 10 min. The crystal violet was removed and cells were washed with tap water until clear. Colonies containing ⩾50 cells were counted. For each experiment, four culture dishes were performed and experiments were carried out in triplicate. Surviving fraction was calculated from the number of colonies formed in the cerulenin-treated dishes compared with the number formed in the untreated control, where plating efficiency is defined as the percentage of cells plated that form colonies, and surviving fraction=number of colonies formed/(number of cells plated × plating efficiency).

### Irradiation

Cells were irradiated with a range of single doses of 0–8 Gy of *γ* rays using a ^137^Cs self-shielded irradiator at a dose rate of 4.0 Gy min^−1^. All irradiations were performed at room temperature; control cells received sham irradiation. After irradiation, the culture plates were returned to the incubator and maintained at 37°C for 96 h. Cell viability was assessed using the MTT assay described above.

### Flow cytometric analysis of cell cycle status

Flow cytometric evaluation of the cell cycle was performed using a modified protocol (Amant *et al*, 2003). Briefly, 5 × 10^5^ cells were plated on 100 mm dishes in medium complemented with 10% FBS overnight. The culture medium was then replaced using serum-free medium for 24 h and the cells treated with 0–10 *μ*g ml^−1^ cerulenin for 24 h. Cells were then trypsinised and collected into ice-cold PBS, and fixed with ice-cold 70% ethanol in PBS for at least 24 h at 4°C. Cells were then stained using 1 ml propidium iodide (PI) solution (20 mg l^−1^ PI and 20 mg l^−1^ RNase in PBS) for 3 h and read on a flow cytometer (Beckman-Coulter, Fullerton, CA, USA).

### Fatty acid synthase shRNA constructs

The nucleotide targets of the FAS coding sequence were selected based on previously published experiments (De Schrijver *et al*, 2003) and computer-based target analysis using siRNA Target Finder (Ambion Inc., Austin, TX, USA). Two 19 base pair siRNA oligonucleotides were used in our study according to siRNA selective guidelines (Ambion Inc.). The first oligonucleotide was AACCCTGAGATCCCAGCGCTG, corresponding to the nucleotides 1212–1231 of human FAS; the second oligonucleotide was AAGCAGGCACACACGATGGAC, corresponding to the nucleotides 329–348. The two target sequences were aligned to the human genome in a BLAST search to eliminate those with significant homology to other genes. A scrambled nucleotide sequence was designed and used as the negative control. The loop sequences and overhangs were added to form the short hairpin constructs. Then, the short hairpin RNA (shRNA) encoding oligonucleotides were cloned into the *Bam*H1 and *Hin*dIII restriction sites downstream of the H1 promoter in pSilencer 3.0-H1 according to the manufacturers’ instructions (Invitrogen, Grand Island, NY, USA).

### Transient transfection

Transient transfections were performed using Lipofectamine according to the manufacturer's instructions (Invitrogen). Briefly, 5 × 10^6^ cells were plated in a 60 mm dish supplied with 5 ml media for 24 h. Cells were then washed twice with serum- and antibiotic-free DMEM medium. Four micrograms of FAS RNAi plasmids/scrambled negative control plasmid and 20 *μ*l Lipofectamine were premixed for 20 min and applied to cells in 4 ml serum- and antibiotic-free DMEM medium. After 4 h, the serum- and antibiotic-free DMEM medium was replaced with 5 ml of complete medium.

### Stable transfection

U251 cells were grown on 60 mm dishes for 24 h and were then transfected with 2 *μ*g of the endoplasmic reticulum (ER)-targeted GFP-Bcl-2 expression vector, wild-type (WT) GFP-Bcl-2 expression vector, or empty vector (gift of Dr Clark W Distelhorst, Case Western Reserve University Medical School and University Hospitals of Cleveland, Cleveland, OH, USA) using Lipofectamine plus reagent according to manufacturer's protocol (Life Technologies). The positive transfected cells were selected in the presence of the neomycin-analogue G418 (800*μ*g ml^−1^).

### Dominant-negative FADD expression

Recombinant doxycycline (Dox)-regulated YFP and dominant-negative YFP-FADD-DN adenoviruses were made using the AdenoX Tet-off kit from Clontech (Palo Alto, CA, USA). Viruses were produced according to the manufacturer's instructions. Dominant-negative adenovirus FADD-DN or adenovirus YFP (empty vector) were co-infected into U251 and SNB-19 glioma cells with a Tet repressor virus. Once FAD-DN was expressed at high levels, as demonstrated by robust YFP fluorescence, cells were treated as required.

### Statistical analysis

Statistical analysis was carried out using one-sample Student's *t*-test to compare differences between the treated cells and their appropriate controls. A *P*-value of <0.05 was considered significant.

## RESULTS

### Fatty acid synthase is overexpressed in human and rat glioma cell lines as well as in human glioma tissue samples

**Figure 1:**
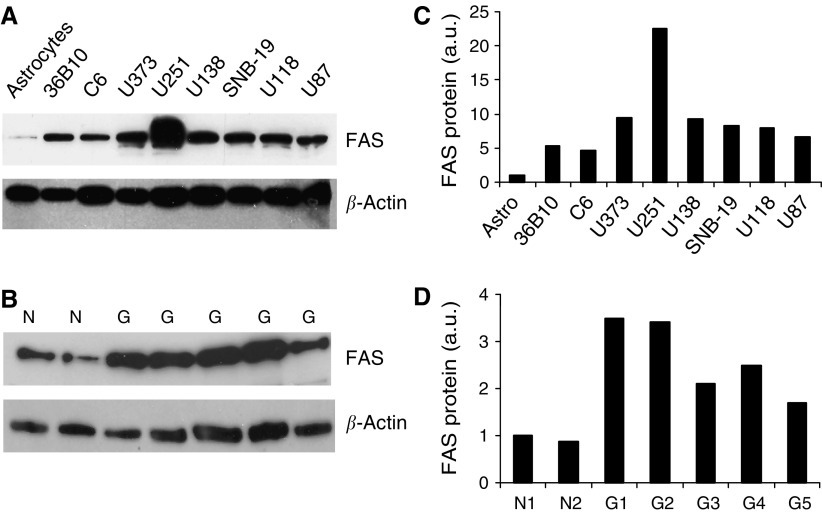
Fatty acid synthase is overexpressed in human and rat glioma cell lines and in human glioma tissue compared with primary rat astrocytes and normal human brain tissue, respectively. U138, U251, U373, SNB-19 human, and C6 rat glioma cells, as well as primary rat astrocytes were grown on 100 mm dishes until 90% confluent. The protein level of FAS was analysed by Western immunoblotting using a monoclonal mouse anti-FAS antibody; *β*-actin protein level was used as a loading control. (**A**) A representative Western Blot indicating the differential expression of FAS in rat and human glioma cell lies as well as primary rat astrocytes. (**C**) The mean densitometric values obtained from two independent experiments. (**B**) A Western blot of lysates obtained from normal human brain as well as several human glioma tissue samples. (**D**) The densitometric analysis of these blots.

To determine if there is differential expression in glioma cells compared with normal brain cells, we measured FAS protein levels in six human glioma cell lines, U251, U373, U138, U118, U87, and SNB-19 and in two rat glioma cell lines, C6 and 36B10, as well as in primary rat astrocytes. Cells were cultured and lysed in 500 *μ*l lysis buffer containing 50 mM Tris-HCl (pH 7.4), 150 mM NaCl, 1% NP-40, 0.25% Na-deoxycholate, and 1 mM of EDTA, PMSF, Na_3_*V*O_4_ and NaF, and proteinase inhibitors. Fatty acid synthase protein levels were analysed by Western blotting. As shown in Figure 1A, a low level of FAS protein was detected in normal primary astrocytes. In contrast, markedly elevated levels of FAS protein were observed in the entire human and rat glioma cell lines. Densitometric analysis revealed 4- to 20-fold increases in FAS expression in the glioma cell lines in comparison with that seen in primary rat astrocytes (Figure 1B). Elevated levels of FAS protein, ranging from 2- to 3.5-fold increases, were also observed in lysates of human glioma tissue samples compared with those obtained from normal human brain (Figure 1C and D).

### Treating glioma cells with the FAS inhibitor cerulenin leads to inhibition of fatty acid synthesis

**Figure 2:**
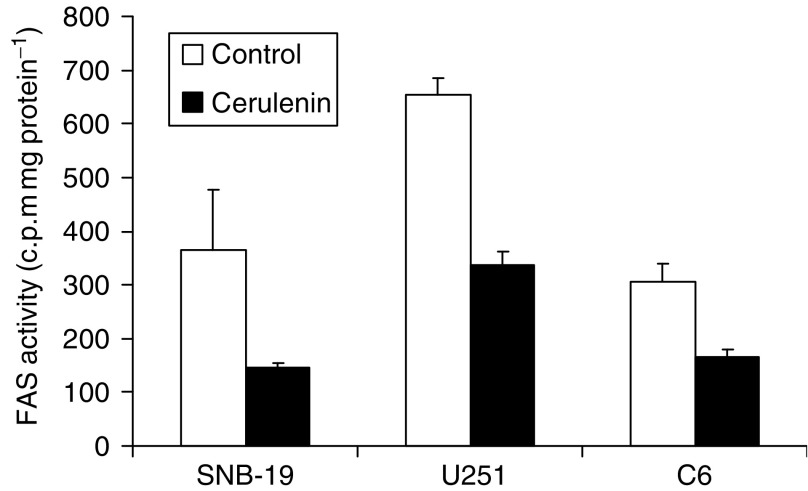
Treating glioma cells with the FAS inhibitor cerulenin leads to inhibition of fatty acid synthesis. U251 and SNB-19 human and C6 rat glioma cells were treated with 5 *μ*g ml^−1^ cerulenin for 2 h. Fatty acid synthesis activity was then measured using the ^14^C-labelled acetate assay. Lipids were extracted and cerulenin-induced inhibition of labelled acetate incorporation into lipid was measured using liquid scintillation counting. Mean±s.e.; *n*=3; ^*^P<0.05.

Cerulenin, a potent noncompetitive pharmacological inhibitor of FAS, binds covalently to the active site of the condensing enzyme region, inactivating a key enzyme step in fatty acid synthesis. To confirm the inhibitory effect of cerulenin on FAS in glioma cells, fatty acid synthesis activity was measured using the ^14^C-labelled acetic acid method (Rashid *et al*, 1997). U251 and SNB-19 human glioma as well as C6 rat glioma cells were incubated with or without cerulenin for 2 h. After labelling with ^14^C-actic acid, the total lipid was extracted and the amount of ^14^C incorporated in the extracted total lipids was determined using liquid scintillation counting. As shown in Figure 2, endogenous fatty acid synthesis in cerulenin-treated glioma cells decreased by approximately 50% in SNB-19, U251, and C6 cells compared with untreated controls. Fatty acid synthase activity was not detectable in primary rat astrocytes (data not shown).

### Incubating glioma cells with cerulenin leads to selective time- and dose-dependent decreases in glioma cell viability and survival; normal astrocytes appear unaffected

**Figure 3:**
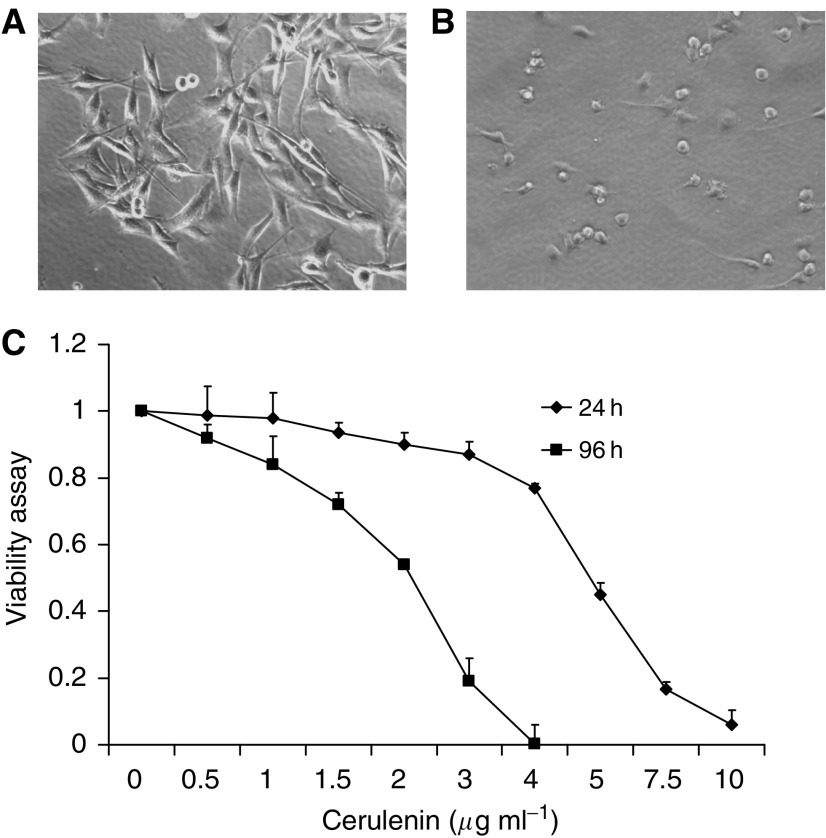
Cerulenin reduces U251 cell viability in a time- and dose-dependent manner. 5 × 10^4^ or 5000 U251 human glioma cells were seeded into 24-well plates and treated with 0–10 *μ*g ml^−1^ cerulenin for 24 and 96 h, respectively. Cell viability was assessed using the MTT assy. (**A**) The morphological appearance of DMSO-treated control cells observed 24 h after treatment. (**B**) Incubating U251 glioma cells with cerulenin for 24 h resulted in the cells adopting a rounded up appearance. (**C**) Incubating U251 glioma cells with cerulenin cells led to a time- and dose-dependent reduction in cell viability. Mean±s.e.; *n*=3.

**Figure 4:**
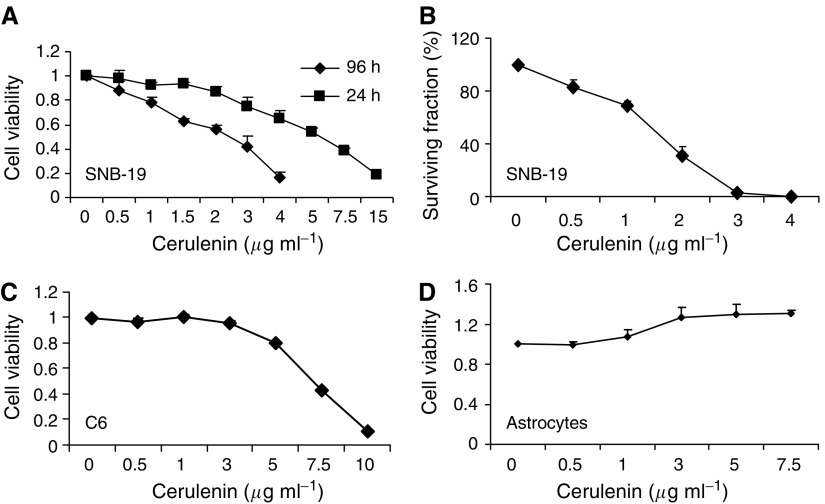
Cerulenin selectively reduces glioma cell viability and clonogenic survival; astrocytes are unaffected. 5 × 10^4^ and 5000 SNB-19 human glioma cells were seeded into 24-well plates and treated with 0–10 *μ*g ml^−1^ cerulenin for 24 and 96 h, respectively. Cell viability was assessed using the MTT assay. To determine clonogenic survival, 500–2000 cells were seeded into 60 mm dishes and treated with 0–10 *μ*g ml^−1^ cerulenin for 10–15 days. Cells were fixed and stained with violet blue and colonies were counted using a colony counter. (**A**) Incubating SNB-19 cells with cerulenin led to a time- and dose-dependent reduction in cell viability. (**B**) A dose-dependent cerulenin-mediated reduction in clonogenic survival. (**C**) Incubating C6 rat glioma cells with cerulenin led to a time- and dose-dependent reduction in cell viability. In contrast, (**D**) shows that incubating primary rat astrocytes with 0.5–7.5 *μ*g ml^−1^ cerulenin for 24 h did not affect cell viability.

To demonstrate whether cerulenin is selectively cytotoxic to glioma cells, we measured the effects of cerulenin on glioma cells and primary rat astrocytes using the MTT and/or clonogenic assay. U251, SNB-19, U87, U118 human, and C6 rat glioma cells were treated with 0–15 *μ*g ml^−1^ cerulenin for 24 and 96 h. We observed rounded up cells following incubation with higher doses (Figure 3A and B) in 24 h and cell death. As shown in Figure 3C, the mean IC_50_ values for U251 cells were 4.7 and 2.3 *μ*g ml^−1^ at 24 and 96 h, respectively. SNB-19 glioma cells showed a similar cytotoxic response to treatment with cerulenin; the mean IC_50_ values for SNB-19 cells were 5.3 and 2.6 *μ*g ml^−1^ at 24 and 96 h, respectively (Figure 4A). Moreover, treatment with cerulenin led to a significant reduction in glioma cell clonogenic survival; incubating SNB-19 cells with 3 *μ*g ml^−1^ cerulenin reduced clonogenic cell survival by 97% compared to non-treated controls (Figure 4B). We also compared the cytoxicity of cerulenin to C6 rat glioma cells and to normal primary rat astrocytes. After treatment with 10 *μ*g ml^−1^ of cerulenin for 24 h, glioma cell viability was reduced to less than 10%; the mean IC_50_ value was 6.5 *μ*g ml^−1^ (Figure 4C). In contrast, primary astrocytes appeared resistant to cerulenin treatment. Incubating astrocytes with 0.5–7.5 *μ*g ml^−1^ cerulenin for 24 h failed to affect cell viability (Figure 4D).

### Cerulenin-induced cell death occurs via an apoptotic mechanism

**Figure 5:**
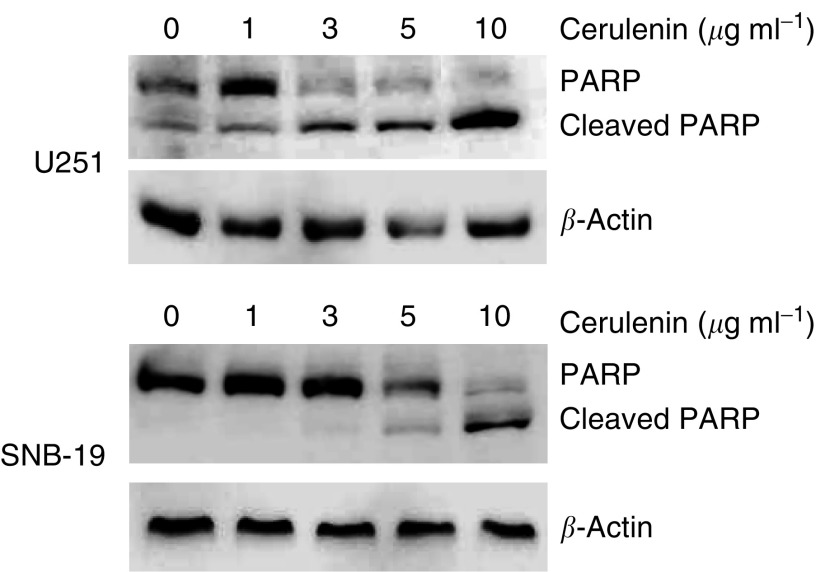
Cerulenin induces PARP cleavage in human glioma cells. U251 and SNB-19 cells were treated with 0–10 *μ*g ml^−1^ cerulenin for 24 h and then collected and lysed using lysis buffer. Poly(ADP-ribose) polymerase, cleaved PARP, and *β*-actin protein levels were analysed using Western blot. Representative Western blots show that cerulenin treatment was associated with dose-dependent increases in cleavage of PARP in both U251 (**A**) and SNB-19 (**B**) glioma cells. Data represent results of two independent experiments.

Poly(ADP-ribose) polymerase, an enzyme catalysing the poly(ADP-ribosyl)ation of various nuclear proteins with NAD, has been suggested to contribute to cell death by depleting the cell of NAD and ATP (Berger *et al*, 1983). Poly(ADP-ribose) polymerase cleavage is a well-established marker for apoptosis. To determine whether apoptosis is involved in cerulenin-induced cell cytotoxicity, we measured PARP cleavage as well as assessing morphological changes. As noted above, cell morphology was changed; cells appeared rounded up after incubation with cerulenin, resulting in cell death. To determine PARP cleavage, U251 and SNB-19 cells were treated with 0–10.0 *μ*g ml^−1^ cerulenin for 24 h and Western blot analysis was used to study the alterations in protein level of PARP in treated and non-treated glioma cells. Treatment with cerulenin led to a dose-dependent increase in cleaved PARP protein in both glioma cell lines (Figure 5).

### Overexpressing Bcl-2 leads to inhibition of cerulenin-mediated cytotoxicity

**Figure 6:**
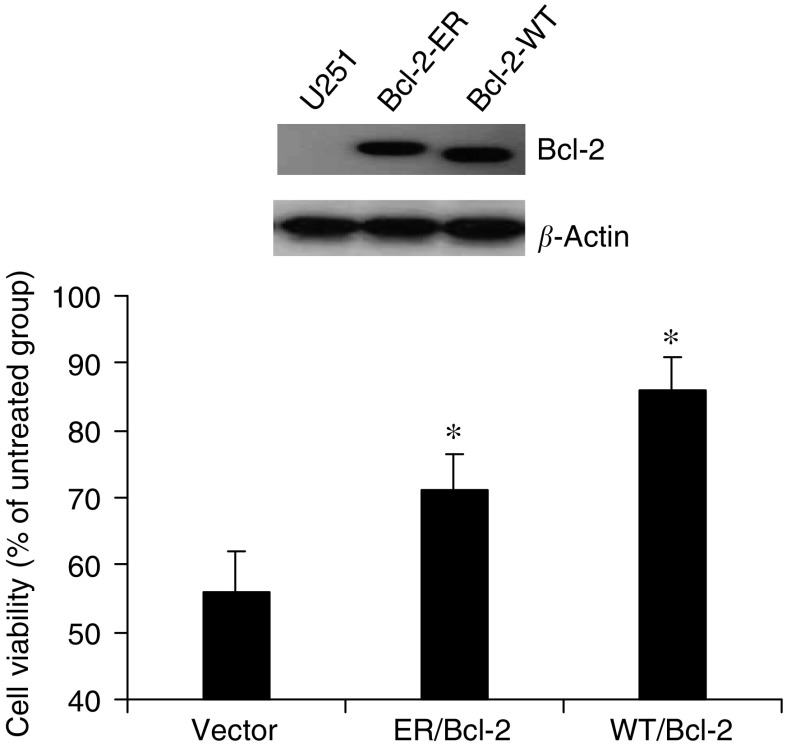
Bcl-2 overexpression protects U251 cells from cerulenin-induced cytotoxicity. U251 cells stably transfected with empty vector, ER-targeted vector, or WT Bcl-2 vector showed a marked increase in levels of Bcl-2 immunoreactive protein (**A**). Cells were treated with 5*μ*g ml^−1^ cerulenin for 24 h. Cell viability was then assessed using the MTT assay (**B**). Mean±s.e.; *n*=4, *P*<0.05.

Overexpression of the antiapoptotic protein Bcl-2 has been shown to inhibit cell death induced by several apoptotic signals and/or mediators (Wang *et al*, 2001a). To determine if overexpressing Bcl-2 would result in a similar inhibition of cerulenin-mediated apoptosis, U251 cells were stably transfected with either WT Bcl-2, ER-targeted Bcl-2, or empty vector. As shown in Figure 6A, transducing cells with Bcl-2 led to a marked increase in protein levels compared with the vector-transduced cells. U251 cells were then treated with 5 *μ*g ml^−1^ of cerulenin for 24 h, and cell viability assessed using the MTT assay. As noted previously, incubating U251 cells stably transfected with vector control alone with 5 *μ*g ml^−1^ cerulenin was associated with a reduction in cell viability to 55.5+3.4% of controls. Overexpressing ER/Bcl-2 and WT/Bcl-2 increased cell viability to 71.0+3.2 and 85.9±2.8%, respectively (*P*<0.05; Figure 6).

### The death receptor FADD pathway is not involved in cerulenin-induced apoptosis

**Figure 7:**
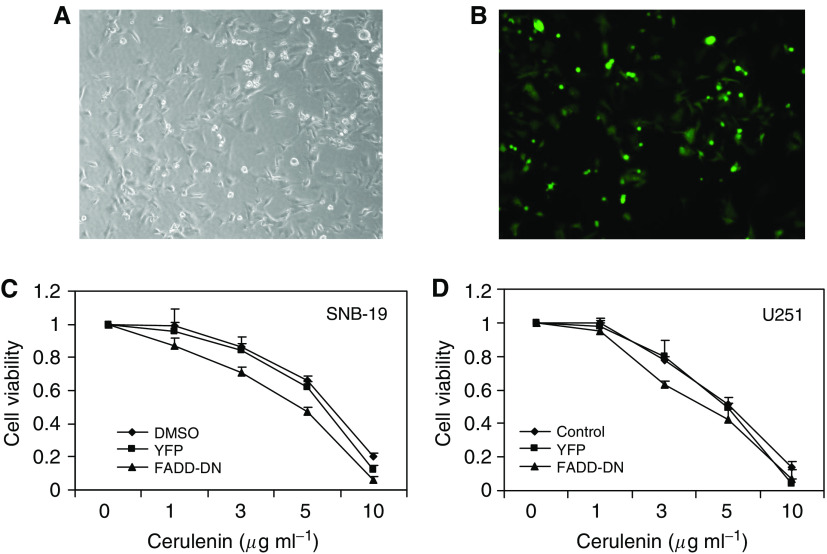
The death receptor FADD pathway is not involved in cerulenin-induced apoptosis. U251 and SNB-19 human glioma cells were co-infected with adenoviruses expressing YFP or TFP-tagged FADD-DN. (**A**) U251 cells treated with DMSO, (**B**) U251 cells co-infected with YFP-FADD-DN. Cells were then treated with 0–10 *μ*g ml^−1^ cerulenin for 24 h and cell viability was then assessed using the MTT assay. Co-infection with YFP or YFP-FADD-DN failed to inhibit the cerulenin-mediated glioma cell cytotoxicity.

FADD-DN is a dominant-negative form of the FADD protein that blocks all known death receptor-induced apoptotic pathways. Activation of the death receptor pathway has been implicated in the mechanism of various chemotherapeutic agents that can work by upregulating death receptor levels or by raising autocrine production of death ligand. To test if such pathways contribute to cerulenin-induced apoptosis, U251 and SNB-19 cells were co-infected with adenoviruses expressing YFP or YFP-tagged FADD-DN. Fluorescence could be observed in infected cells (Figure 7B). After co-infection for 48 h, uninfected control cells, YFP-expressing cells, and YFP-FADD-DN-expressing cells were treated with 0–10 *μ*g ml^−1^ cerulenin for 24 h and the MTT assay was performed to measure cell viability. As shown in Figure 7C and D, no significant differences in cell viability were found in the various groups, indicating that the death receptor pathway is not associated with FAS inhibition-induced cell apoptosis.

### Treating human glioma cells with cerulenin leads to an S-phase cell cycle block

**Figure 8:**
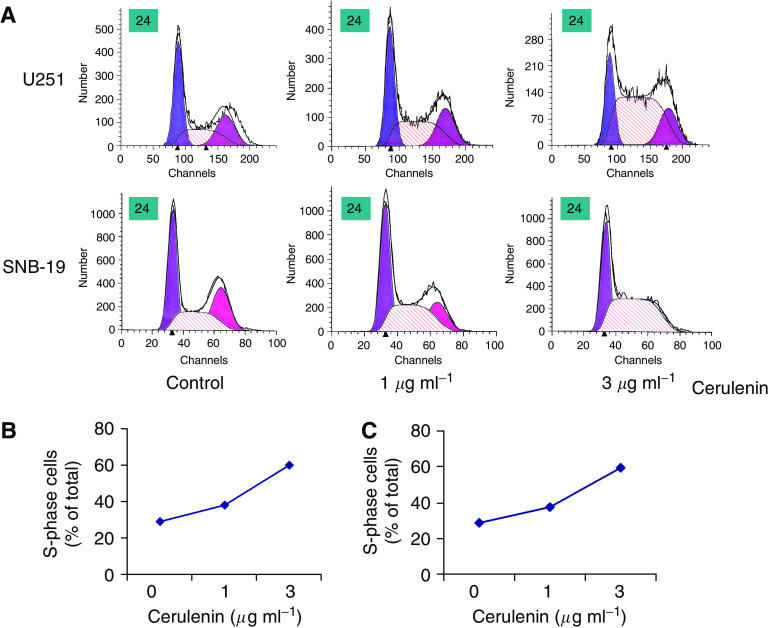
Treating human glioma cells with cerulenin leads to a dose-dependent accumulation of cells in S phase. U251 and SNB-19 human glioma cells were treated with DMSO, 1 *μ*g ml^−1^ cerulenin, or 3 *μ*g ml^−1^ cerulenin for 24 h. The cells were then harvested, fixed, stained with PI, and analysed by flow cytometry. (**A**) A representative flow cytometry experiment; (**B**) and (**C**) the mean data obtained from the results of two independent experiments. These data indicate that incubating U251 and SNB-19 cells with cerulenin led to a dose-dependent increase in S-phase cell accumulation.

We hypothesised that the cerulenin-induced cell growth inhibition and death might involve cell cycle arrest. To test our hypothesis, we performed flow cytometry analysis. U251 and SNB-19 human glioma cells were treated with DMSO, 1 or 3 *μ*g ml^−1^ cerulenin for 24 h. The cells were then collected and stained with PI for flow cytometry analysis. Representative cell cycle profiles are shown in Figure 8. After treatment with cerulenin for 24 h, the number of U251 cells in S phase increased markedly to 58% of the total cell population compared with controls (23%). A similar increase in the % of cells in S phase was seen 24 h after treatment of SNB-19 cells with cerulenin (Figure 8). These results suggest that cerulenin-mediated FAS inhibition induced an S-phase block in glioma cells.

### Pretreating glioma cells with cerulenin does not alter radiosensitivity

**Figure 9:**
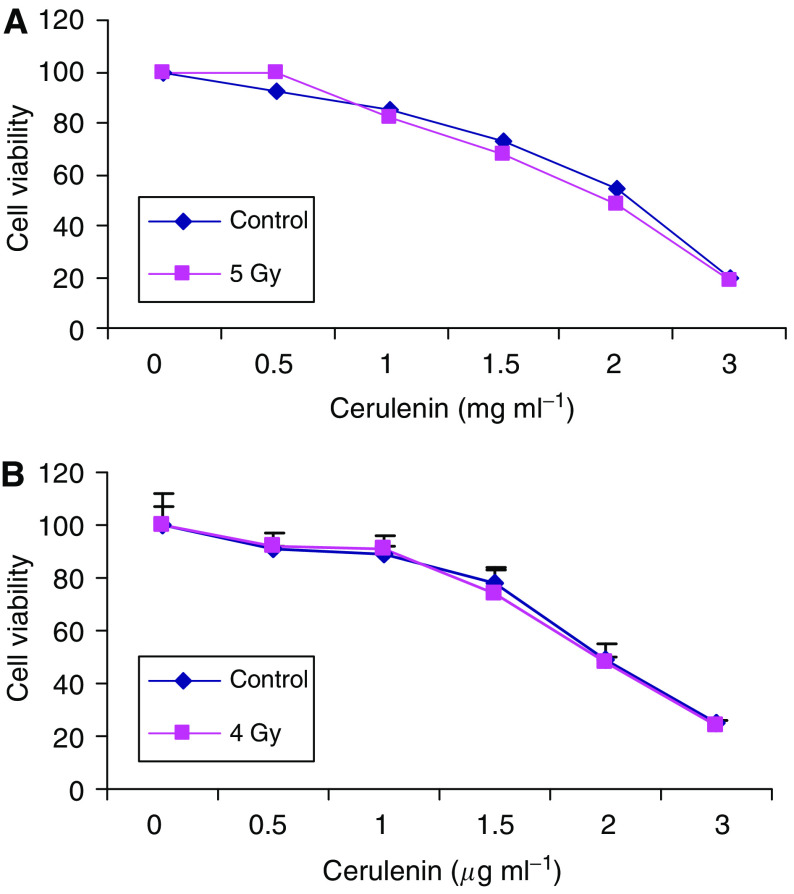
Treating human glioma cells with cerulenin fails to alter their radiosensitivity. SNB-19 human glioma cells were treated with 0.5–4.0 *μ*g ml^−1^ cerulenin for 2 h, control cells received DMSO. The cells then received either *γ* irradiation (single dose of 4 or 5 Gy *γ* rays [▪]) using a ^137^Cs irradiator or sham irradiation (⧫), incubated for 96 h, and then viability was assessed using the MTT assay.

Cell radiosensitivity is cell cycle dependent; cells present in the S phase are relatively radioresistant as compared with cells in other phases of the cell cycle (Pawlik and Keyomarsi, 2004). As treating glioma cells with cerulenin induced an S-phase block, we hypothesised that irradiating glioma cells in the presence of cerulenin would lead to either no effect or increased radioresistance. As shown in Figure 9A, there was no difference in viability between glioma cells treated with 0.5–4.0 *μ*g ml^−1^ of cerulenin 2 h before irradiation with a single dose of 5 Gy of ^137^Cs *γ* rays when compared with glioma cells treated with radiation alone. Increasing the time the cells were treated with cerulenin to 8 h before irradiation similarly failed to alter glioma cell radiosensitivity. Irradiating cerulenin-treated cells with single doses of 2, 4, or 8 Gy had no affect on glioma cell radiosensitivity. Figure 9B shows data for glioma cells treated with cerulenin 8 h before a single dose of 4 Gy; a similar response was seen after doses of 2 and 8 Gy (data not shown).

### Fatty acid synthase shRNA transfection leads to downregulation of FAS expression and enzymatic activity that is associated with decreased glioma cell viability

**Figure 10:**
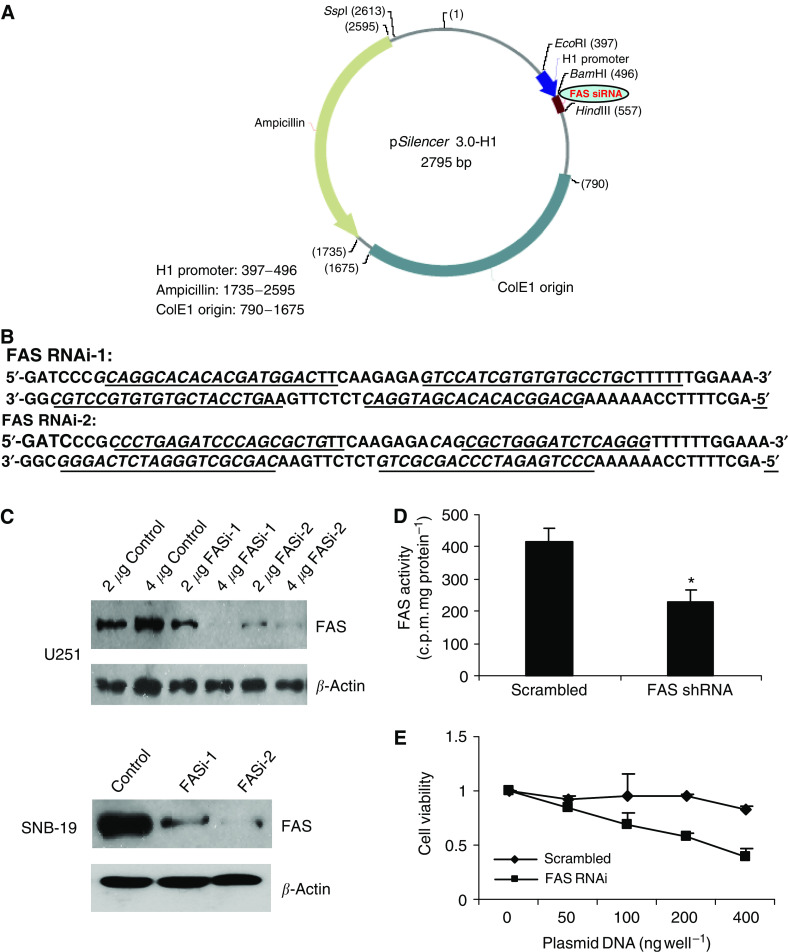
Fatty acid synthase shRNA transfection downregulates FAS expression and inhibits glioma cell growth. Sense and antisense oligonucleotides encoding the shRNAs (**B**) were cloned into the *Bam*H1 and *Hin*dIII restriction sites downstream of the H1 promoter in pSilencer 3.0H-1 (**A**). U251 and SNB-19 cells were seeded into 60 mm dishes for 24 h and transfected with 2 and 4 *μ*g FAS shRNA DNA for a further 72 h. Cells were then lysed and FAS protein expression analysed using Western blot analysis. As shown in (**C**), FAS shRNA1 and FAS shRNA2 reduced markedly FAS protein levels; FAS enzymatic activity was also significantly reduced (**D**). Control cells transfected with a scrambled shRNA showed no change in FAS expression. As shown in (**E**), cell viability was significantly reduced in U251 glioma cells transfected with FAS RNAi.

To confirm that the effects of cerulenin were due to inhibition of FAS, we used RNAi to knock down FAS protein levels. To identify effective RNAi against FAS expression, we tested two different plasmid-based hairpin constructs of FAS RNAi; a scrambled sequence was used as the negative control. The shRNAs encoding oligonucleotides are shown in Figure 10B. They were cloned into the *Bam*H1 and *Hin*dIII restriction sites downstream of the H1 promoter in pSilencer 3.0-H1 (Figure 10A). U251 and SNB-19 cells were seeded into 60 mm dishes for 24 h and transfected with 2 and 4 *μ*g FAS shRNA DNA for a further 72 h. Cells were then lysed and FAS protein expression analysed using Western blot analysis. As shown in Figure 10C, scrambled shRNA (control) had no effect on FAS expression. In contrast, FAS shRNA1 and FAS shRNA2 reduced significantly FAS protein levels. Moreover, this reduction in FAS protein was associated with a significant (*P*<0.05) reduction in FAS enzymatic activity (Figure 10D). To test the effects of RNAi-mediated FAS silencing on glioma cell growth, SNB-19 cells were transiently transfected with a range of concentrations of plasmid DNA of scrambled shRNA and FAS shRNA for 72 h. Cytotoxicity was determined using the MTT assay. As shown in Figure 10E, cell viability was dose-dependently reduced in glioma cells transfected with FAS RNAi. These data confirm the effect of cerulenin as being FAS-specific.

## DISCUSSION

Our data reveal, for the first time, that the levels of FAS expression observed in a variety of rat and human glioma cell lines and several human glioma tissue samples are higher compared to their normal cell counterpart, the astrocyte, as well as normal human brain tissue, respectively. Mammalian FAS is a multifunctional enzyme complex that catalyses the synthesis of palmitate from acetyl-CoA and malonyl-CoA with NADPH as a provider of reducing equivalents (Kuhajda, 2006). Humans take up large amount of proteins and lipids through their daily diet. Therefore, the levels of FAS in most tissues are low. In human tissue, FAS is distributed in cells involved in lipid metabolism and in hormone-sensitive cells, such as adipocytes, hepatocytes, sebaceous glands, and type II alveolar cells (Weiss *et al*, 1986). High FAS activity was observed in foetal brain tissue and gradually declined following aging (Salles *et al*, 2002). FAS mRNA level in 20-day-old rat brain was about 20% compared with that measured in 5-day-old brain (Garbay *et al*, 1997; Muse *et al*, 2001). In the adult rat brain, immunohistochemistical analysis for FAS revealed positive staining cells in cortical neurons of the frontooccipital lobes; only weak staining was observed in astrocytes (Kusakabe *et al*, 2000).

Glioma cell FAS expression has not been reported previously. We found a marked differential expression of FAS in glioma and normal astrocytes. A similar upregulation of FAS expression was noted in lysates of human glioma samples compared with lysates of normal human brain tissue. Administration of the FAS inhibitor cerulenin led to significant time- and dose-dependent decreases in glioma cell viability and survival. Low doses of cerulenin caused cell cycle arrest, whereas higher doses led to cytotoxicity. In contrast, normal astrocytes were unaffected. These findings suggest that FAS might be a novel target for antiglioma therapy.

The mechanism(s) underlying malignant cell cytotoxicity following FAS inhibition has been studied in several cancer cell lines. FAS inhibition can cause accumulation of malonyl-CoA, which leads to inhibition of carnitine palmitoyltransferase-1 and, indirectly, the fatty acid oxidation pathway (Thupari *et al*, 2001). Rapid accumulation of malonyl-CoA was observed after FAS inhibition (Kuhajda *et al*, 2000). Blocking the accumulation of malonyl-CoA using 5-(tetradecyloxy)-2-furoic acid, an inhibitor of acetyl-CoA carboxylase, reduced significantly cerulenin- and C75-induced cytotoxicity and inhibition of breast tumour growth. Apoptosis appeared to play a major role in this cell death. In our experiments, we demonstrated clearly that PARP cleavage, an apoptotic marker, was induced in cerulenin-treated U251 and SNB-19 cells; this was associated with significant cytotoxicity. Heiligtag *et al* (2002) reported that cerulenin is an effective inducer of apoptosis in WT or mutant p53 neuroblastoma, melanoma, and colon carcinoma cell lines; normal human cells were resistant to cerulenin-induced apoptosis. Further, apoptosis was mediated both by overexpression of proapoptotic Bax, in a p53-independent manner, and by a rapid release of mitochondrial cytochrome *c*, leading to activation of caspase 3 and 9. Data presented in the current studies confirm a P53-independent apoptotic response in glioma cells following FAS inhibition; similar glioma cell cytotoxicity was observed in either P53 WT (U87 and U118) or in P53 mutant glioma cell lines (U251, SNB-19, U138, and C6).

Bcl-2, a protooncogene that enhances cell survival by inhibiting apoptosis (Vaux *et al*, 1988), is localised in the ER, the outer mitochondrial membrane, and the nuclear envelope (Akao *et al*, 1994). Stable expression of Bcl-2 or Bcl-2 targeted to the ER has been shown to inhibit apoptosis (Wang *et al*, 2001a). Similarly, we observed that stable expression of Bcl-2-targeted to the ER or WT Bcl-2 expressed not only in the ER but also in the mitochondria and nuclei, reduced cerulenin-mediated glioma cell death. These data provide additional support for the hypothesis that cerulenin-mediated toxicity is due, in part, to an increase in apoptotic cell death. To investigate further the apoptotic response, the role of the death receptor pathway in cerulenin-mediated apoptosis was evaluated by infecting glioma cells with a FADD-DN adenovirus. In agreement with the previously suggested mechanism involving cytochrome *c* release (Heiligtag *et al*, 2002) FADD-DN, which inhibits all the known death receptor pathways, had no effect on cerulenin-induced glioma cell death.

Cerulenin- and C75-mediated alterations in cell cycle progression have been observed in a variety of cancer cell lines. Although FAS inhibition can lead to a block in the cell cycle before G_1_ (Kuhajda *et al*, 2000), there are data that support an S-phase arrest in breast cancer cells (Zhou *et al*, 2003) and in colon carcinoma cells (Pizer *et al*, 2001). In the current study, we demonstrated a marked, dose-dependent S-phase block at 24 h after treatment. The mechanism(s) of FAS inhibition-induced cell cycle arrest is still not clear. *De novo* fatty acid synthesis by tumour cells accounted for more than 93% of total lipid fatty acids in an experimental tumour model, indicating that endogenous fatty acid synthesis could be a significant source of fatty acids for tumour cell growth and survival (Ookhtens *et al*, 1984). Most of the fatty acids produced by tumour cells are incorporated into membrane phospholipids, and phospholipid synthesis is inhibited when fatty acid synthesis is inhibited (Pizer *et al*, 1996; Jackowski *et al*, 2000). Phospholipid biosynthesis is greatest during the G_1_ and S phases, with doubling of the membrane mass occurring during S phase in preparation for cell division (Jackowski, 1994). Thus, cells in S phase should be most sensitive to changes in phospholipid metabolism. In contrast, cells in S phase are relatively radioresistant; the expected outcome would be either no effect or increased radioresistance. Indeed, as predicted, combining cerulenin treatment with ionising radiation did not alter glioma cell radioresistance.

We used selective gene silencing by siRNA (Zamore *et al*, 2000) to confirm the role of FAS inhibition in glioma cell cytotoxicity. RNAi-induced knockdown of FAS that led to a significant reduction in FAS enzymatic activity resulted in a dose-dependent reduction in glioma cell viability, confirming similar gene silencing approaches with RNAi described in human LNCaP prostate cancer cells (De Schrijver *et al*, 2003).

In conclusion, our studies demonstrate that (1) human and rat glioma cell lines express high levels of FAS compared with normal brain astrocytes; high levels of FAS were also seen in human glioma tissue compared with normal human brain, (2) FAS inhibition is selectively cytotoxic to glioma cells; normal astrocytes are unaffected; and (3) both cytotoxic and cytostatic effects contribute to the cerulenin-mediated effects in glioma cells. These findings support the hypothesis that FAS might be a novel target for antiglioma therapy.
